# Circulating MicroRNAs as Potential Molecular Biomarkers in Pathophysiological Evolution of Pregnancy

**DOI:** 10.1155/2016/3851054

**Published:** 2016-07-17

**Authors:** Dragos Cretoiu, Jiahong Xu, Junjie Xiao, Nicolae Suciu, Sanda Maria Cretoiu

**Affiliations:** ^1^Division of Cellular and Molecular Biology and Histology, Carol Davila University of Medicine and Pharmacy, 050474 Bucharest, Romania; ^2^Victor Babeș National Institute of Pathology, 050096 Bucharest, Romania; ^3^Department of Cardiology, Tongji Hospital, Tongji University School of Medicine, Shanghai 200065, China; ^4^Regeneration and Ageing Lab, Experimental Center of Life Sciences, School of Life Science, Shanghai University, Shanghai 200444, China; ^5^Department of Obstetrics and Gynecology, Polizu Clinical Hospital, Carol Davila University of Medicine and Pharmacy, 011062 Bucharest, Romania; ^6^Alessandrescu-Rusescu National Institute of Mother and Child Health, 020395 Bucharest, Romania

## Abstract

MicroRNAs represent nonprotein coding small RNA molecules that are very stable to degradation and responsible for gene silencing in most eukaryotic cells. Increased evidence has been accumulating over the years about their potential value as biomarkers for several diseases. MicroRNAs were predicted to be involved in nearly all biological processes from development to oncogenesis. In this review, we address the importance of circulating microRNAs in different conditions associated with pregnancy starting with the implantation period to preeclampsia and we shortly describe the correlation between placental circulating miRNAs and pregnancy status. We also discuss the importance of microRNAs in recurrent abortion and ectopic pregnancy.

## 1. Introduction

MicroRNAs (miRNAs) are short, single-stranded RNA (19–25 nucleotide long) nonprotein coding genes able to recognize complementary messenger RNAs (mRNAs), acting as master gene regulators by repressing mRNA translation or by mRNA degradation (reviewed in extenso by [1, 2]). MicroRNAs proved to be involved in numerous biological processes from development to oncogenesis [3, 4].

In biomedical research, miRNAs are gaining more and more importance as novel biomarkers for diagnosis, prediction, prognosis, and reaction to therapy. Lately, it became evident that circulating miRNAs might be used as biomarkers for a great number of diseases and in fact represent the forthcoming expectation for a noninvasive diagnostic screening [5, 6]. Nowadays miRNA-bioinformatics tools and databases are used to cope with the huge amount of information since in humans approximately 3707 novel mature miRNAs were identified [7].

After the discovery of miRNAs in 1993 by Lee et al. [8], significant evidence has accumulated about the physiological relevance of miRNAs. Most of the research was focused on the role of intracellular miRNAs which have been shown to regulate genes involved in differentiation, proliferation, and apoptosis [9]. Over time, it has been shown that miRNAs are ubiquitously present in body fluids and might be the mechanism of genetic exchange between cells in a horizontal manner [10]. Circulating or extracellular miRNAs have been shown to be stable and protected from RNase degradation. This protection is achieved by inclusion either in various (lipo) protein complexes (e.g., HDL, Argonaute protein, and nucleophosmin 1) or in different types of extracellular vesicles [11–15].

Weber et al. divided body fluids into two categories depending on the method of harvesting, (a) without any invasive means: breast milk, colostrum, saliva, seminal fluid, tears, and urine and (b) acquired by invasive procedures—amniotic fluid, cerebrospinal fluid, plasma, bronchial lavage, pleural fluid, and peritoneal fluid, and concluded that “the composition and concentrations of miRNAs are measurably different among them” [16]. Several circulating miRNAs were proposed as diagnostic biomarkers in human diseases and extensive reviews were written, to name but a few [17–21].

Women's reproductive medicine is also encouraged by the possibility to use circulating microRNA profiles (detection and quantification) for the evaluation of the ovarian function, placental function, uterine receptivity, pregnancy detection, embryonic development, and evolution/complication of pregnancy.

In this review, we will characterize the impact of circulating miRNAs as potential molecular biomarkers in the pathophysiological evolution of pregnancy.

## 2. The Biogenesis of MicroRNAs

**Figure 1:**
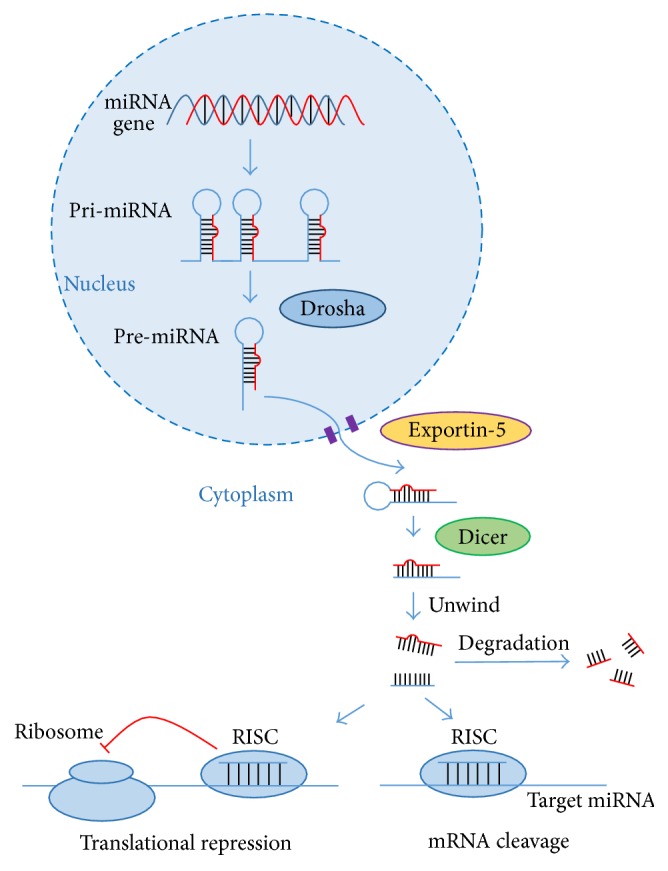
The biogenesis of microRNAs.

miRNA production begins in the nucleus, with RNA polymerase II-mediated transcription. Using genomic DNA as template and generating a long primary miRNA (known as pri-miRNA, which, by folding, becomes a series of hairpin loops), the double-stranded RNA structure of hairpin loop in pri-miRNA can be easily cleaved by Drosha (a double-strand RNase III endonuclease), with the support from DGCR8/pasha [22]. After 70–90 nucleotides, hairpin structure premature miRNA emerges (known as pre-miRNA). The pre-miRNA can bind to nuclear export factor exportin-5 and be exported to the cytoplasm with GTP hydrolysis [23]. In the cytoplasm, pre-miRNA is cleaved by Dicer (a specific double-strand RNA endonuclease), yielding a miRNA:miRNA^*∗*^ duplex about 22 nucleotides in length [24] (Figure 1). In general, only one can recruit Argonaute in RNA-induced silencing complex (RISC) and work as RNA interference. Another is degraded by RISC.

## 3. miRNAs in Embryo-Endometrial Cross Talk at Implantation

A successful implantation depends essentially on timing and a dialogue between the free-floating blastocyst and the receptive endometrium and must be regarded as a multilevel, multiscale integrative approach [25]. It is common knowledge that autocrine, paracrine, and endocrine factors are working closely, coordinating their effects during embryo implantation. It has been suggested that, among this multitude of players, miRNAs might also contribute knowing that their expression throughout the menstrual cycle is sex hormone-dependent. This affirmation is supported by a study of Kuokkanen et al. who compared endometrial samples in the midsecretory phase and in the late proliferative phase. They found that the expression of miR-503 was significantly increased in the late proliferative-phase samples compared to the midsecretory phase samples while the expression level of miR-210, miR-29B, miR-29C, miR-30B, miR-30D, miR193A-3P, miR-200C, and miR-31 was significantly decreased in the late proliferative phase versus midsecretory phase [26]. Several miRNAs were found to be differentially expressed in receptive versus prereceptive human endometria by Altmäe et al. [27]. They concluded that hsa-miR-30b, hsa-miR-30d, hsa-miR-494, and hsa-miR-923 might “play an important role in gene reprogramming at the time of endometrial receptivity” and “could serve as novel biomarkers of fertile receptive endometrium” in the future [27]. Moreover, a prospective analysis conducted on patients who received IVF treatment aimed to determine the effect of higher progesterone level on endometrial receptivity and found four downregulated miRNAs (hsa-miR-451, hsa-miR-424, hsa-miR-125b, and hsa-miR-30b) between normal and elevated progesterone groups that might explain the reduced pregnancy rate in patients with elevated progesterone [28].

There are several studies focusing on the importance of embryo-endometrial cross talk at implantation which seems to be mediated by exosomes released by the endometrium, but none is addressing humans (reviewed by [29]). Exosomes usually contain numerous lipids, proteins, mARNs, and miRNAs [30] and are now considered critical components of uterine luminal fluid [31]. Burns et al. evaluate exosomes in the uterine luminal fluid of sheep and found 81 conserved mature miRNAs emanating from the endometrial epithelia or derived from the conceptus trophectoderm and considered exosomes as essential players important for the establishment and maintenance of pregnancy [31, 32].

Another recent study assessed the role of miR-145 and its target IGF1R in early implantation and showed its involvement in embryo attachment by reducing the level of IGF1R in endometrium and also the importance of the finding in the improvement of pregnancy rates in women with recurrent implantation failure [33].

## 4. miRNAs in Recurrent Abortion

Recurrent abortion is defined as 2 or more consecutive pregnancy losses before the 20th gestational week or spontaneous abortion of a fetus weighing less than 500 g, affecting 1% to 2% of the reproductive age couples worldwide [34]. Recurrent abortion is extremely difficult to treat and novel therapeutic and diagnosis ways are highly needed [34].

Two variant alleles, namely, rs41275794 and rs12976445, in pri-miR-125a have been identified in recurrent abortion in a Chinese-Han population and these variant alleles would lead to the altered production of miR-125a. The decrease of miR-125a caused by these two variant alleles can cause increased LIFR and ERBB2, two target genes of miR-125a, playing critical roles in the embryo implantation and decidualization [35]. Moreover, the rs6505162 C>A in the miR-423 coding region was also identified to be associated with the occurrence of recurrent abortion. The A allele in the polymorphism rs6505162 could more effectively inhibit proliferation-associated 2 group 4 (PA2G4) than the C allele could [36]. Besides, in the Chinese population, a study in the Korean population has also been conducted. They found that miR-196a2CC, miR-499AG+GG, and the miR-196a2CC/miR-499AG+GG combination were associated with recurrent abortion in a Korean population [37].

Human leukocyte antigen- (HLA-) G confers fetal-maternal tolerance and plays an important role in successful pregnancy [38]. miR-133a was reported to be significantly increased in recurrent abortion villi with normal karyotype and HLA-G is a target gene of miR-133a [38]. In addition, miR-34a, miR-155, miR-141, miR-125a, and miR-125b were found to be increased in the recurrent abortion women, while miR-24 was decreased in decidual natural killer cells [39]. PI3K-Akt, MAPK, focal adhesion, T-cell receptor, estrogen, TGF-*β*, and actin cytoskeleton regulation signaling pathways were predicted to be regulated by these miRNAs [39]. Moreover, in the villi of recurrent abortion patients, miR-184, miR-187, and miR-125b-2 were upregulated, while miR-520f, miR-3175, and miR-4672 were downregulated [40]. In the decidua of recurrent abortion patients, miR-517c, miR-519a-1, miR-522, miR-520h, and miR-184 were increased [40]. However, the functional role of these aberrant miRNAs in recurrent abortion is unclear.

A recent work has reported the potential of using plasma miRNAs as biomarkers for recurrent abortion [41]. A total of 27 recurrent abortion patients and 28 normal early pregnancies patients were enrolled at 6–10 weeks of gestation. Based on miRNA microarrays and real-time quantitative reverse transcription polymerase chain reaction analysis, a total of 9 miRNAs were found to be increased while a total of 16 miRNAs were decreased [41]. Further studies confirmed that miR-320b, miR-146b-5p, miR-221-3p, and miR-559 were upregulated, while miR-101-3p was downregulated [41]. This study provides the idea that these circulating miRNAs might be biomarkers of recurrent abortion though the ROC curve has not been performed in the study and the results also need to be validated in an independent cohort.

## 5. miRNAs and Ectopic Pregnancy

Ectopic pregnancy (EP) is defined as conceptus implants outside the endometrial cavity [42, 43]. Although EP occurs in only about 1% to 2% of pregnant women, it is highly detrimental to patients usually leading to tubal rupture and death [44]. Current diagnosis of EP depends on transvaginal ultrasonography and measurement of serum human chorionic gonadotropin (hCG) and progesterone [45]. Owing to the fact that clinical ultrasonography is not always definitive and that serial hCG and/or progesterone assessment is associated with high false-positive and false-negative rates, searching for the novel noninvasive circulating biomarkers for detecting EP is highly important [46].

miRNAs are considered as potential biomarker candidates for multiple pregnancy-associated diseases [47, 48]. Previous studies demonstrated dysregulation of miRNA expressions in early embryonic tissues and in the fallopian tube of women with EP, including Lin28b, let-7, miR-132, miR-145, miR-149, miR-182, miR-196, miR-223, miR-424, and miR-451 [49–51]. However, a limited discovery was obtained with regard to circulating miRNAs as biomarkers for diagnosis of EP [52]. In a multicenter, retrospective, and case-control cohort study, serum levels of hCG, progesterone, and a group of pregnancy-associated miRNAs were analyzed in women with EP, spontaneous abortion (SA), and viable intrauterine pregnancy (VIP) [53]. Data from this study demonstrate that concentrations of serum miR-517a, miR-519d, and miR-525-3p were significantly lower, while the concentration of serum miR-323-3p was higher, in women with EP and SA than in VIP. Among these miRNAs, circulating miR-323-3p has the highest sensitivity when used as a single marker. Furthermore, the combined hCG, progesterone, and miR-323-3p show even higher sensitivity and specificity when compared to each use alone, suggesting that miR-323-3p might be a useful biomarker to improve the diagnosis of EP [53]. In another independent population study, evidence was also gained that circulating level of miR-323-3p could distinguish EP cases from SA cases [54]. Further studies were needed to elucidate the underlying mechanisms by which miRNAs cause the clinical manifestations of EP.

## 6. Placental Circulating miRNAs and Pregnancy Status

Chim et al. showed high maternal plasma concentration of four placental miRNAs (miR-141, miR-149, miR-299-5p, and miR-135b) which fell off in postdelivery plasma indicating a direct correlation with pregnancy status. Moreover, miR-141 concentration increased with gestational age [55]. This preliminary study is suggestive for the potential of miRNAs as molecular markers for pregnancy monitoring and diagnosis.

Kotlabova et al. demonstrated that seven placental specific microRNAs were present in maternal plasma, miR-516-5p, miR-517^*∗*^, miR-518b, miR-520a^*∗*^, miR-520h, miR-525, and miR-526a, and might be pregnancy-associated microRNAs with diagnostic potential [56].

A comparative study determined plasma concentrations of cell-free, pregnancy-associated, placenta-specific microRNAs between nonlabor and labor groups (including 32 women) and found that miR-515-3p, miR-517a, miR-517c, and miR-518b placenta-specific miRNAs in the labor group were significantly higher than those in the nonlabor group before cesarean section and at 24 hours after delivery, respectively [57].

Miura et al. found that there is a direct correlation between maternal plasma levels of cell-free pregnancy-associated placenta-specific miR-515-3p, miR-517a, miR-517c, and miR-518b and placental weight [58]. Hasegawa et al. identified an association between placenta praevia and cell-free pregnancy-associated placenta-specific miRNAs in maternal plasma [59]. They found significantly higher plasma concentrations of cell-free miR-517a and significantly lower plasma concentrations of cell-free miR-518b in the placenta praevia group comparative with the control group and suggested that the circulating level of cell-free miR-517a may be a predictive marker for the risks of bleeding in late pregnancy and of massive hemorrhage at delivery [59].

These studies identified that a number of circulating miRNAs originating in placental trophoblast layer are a trailblazer in the field of identification of noninvasive markers for placental dysfunction. However, many challenges lie ahead before circulating miRNAs will answer actual clinical and therapeutic needs.

## 7. miRNAs in (Pre)Eclampsia

Preeclampsia is defined as a specific condition of late pregnancy, 2nd or 3rd trimester, affecting approximately 2–8% of all pregnancies worldwide and is characterized by maternal high blood pressure and high levels of protein in the urine [60]. Despite its gravity, there are no specific biomarkers predictive of the disorder and only a few studies have implicated an altered miRNA expression. The first study addressing miRNAs importance in preeclampsia dates in 2007 when Pineles et al. analyzed placentas from women with preeclampsia and small-for-gestational age and found different subsets of expressed microRNAs (miR-210, miR-182) in patients with preeclampsia [61]. The following studies support the involvement of placental miRNAs in the setting of preeclampsia and showed upregulated expression of miR-496 and lower expression of miR-15b, miR-181, miR-210, and miR-483–5p [62, 63]. Circulating miRNAs levels in plasma from severe preeclamptic pregnancies were first analyzed by Wu et al. who detected and validated seven elevated miRNAs, miR-24, miR-26a, miR-103, miR-130b, miR-181a, miR-342-3p, and miR-574-5p, as potential markers for diagnosing preeclampsia [64]. Using the next generation sequencing platform of sequencing by oligo ligation detection (SOLiD) and RT-PCR for validation, Li et al. showed in their study that maternal plasma miR-141 and miR-29a were significantly overexpressed, while maternal plasma miR-144 was significantly underexpressed preeclamptic patients compared to normal control suggesting their potential use as preeclampsia biomarkers [65]. Xu et al. carried out a prospective cohort study at gestational weeks 15 to 18 and at term and found low circulating levels of miR-18a, miR-19b1, and miR-92a1 and high levels of miR-210 in preeclamptic patients comparative with normal controls [66]. Luque et al. assessed in a study of the usefulness of circulating microRNAs (miRNAs) as noninvasive molecular biomarkers for early prediction of preeclampsia [67].

A moderate negative correlation between miRNA-942 and the mean arterial pressure was noted and rather weak correlations between miR-143 and the ethnicity, parity, and the mean uterine artery Doppler pulsatility index was found, suggesting that circulating miRNAs have a minor predictive and functional pathophysiological relevance of early preeclampsia at first trimester of pregnancy [67]. Recently, Sandrim et al. compared circulating microRNAs expression profiles between preeclampsia and healthy pregnant women and found increased levels of miR-885-5p in plasma from preeclampsia women which was released into circulation mainly inside exosomes [68]. In another study, the same group also detected increased plasma levels of miR-195-5p in preeclamptic women [69].

As we can ascertain in all the above-mentioned studies, there are conflicting data and very few circulating miRNAs are overlapped. Although these data open perspectives for miRNAs as biomarkers for the prediction of preeclampsia, a large amount of work is required in the future which firstly requires a standardization of methods/techniques used in miRNA profiling.

In conclusion, all these recent data support the evidence that indeed miRNAs are useful candidates in the prediction of the pathophysiological evolution of pregnancy and it is only a matter of time before some of these already described ones will be validated as diagnosis biomarkers.
